# Delayed diagnosis of spontaneous bladder rupture: a rare case report

**DOI:** 10.1186/s12905-018-0616-y

**Published:** 2018-07-11

**Authors:** Pingjin Qiao, Dongmei Tian, Qiao Bao

**Affiliations:** 1 Department of Surgery, Guangdong Women and Children Hospital, Guangzhou, 510010 Guangdong China; 2 Department of Obstetrics, Guangdong Women and Children Hospital, No. 13 Guangyuan West Road, Guangzhou, 510010 Guangdong China; 3 Department of Urology, Guangdong Women and Children Hospital, Guangzhou, 510010 Guangdong China

**Keywords:** Pseudo renal failure, Spontaneous bladder rupture, Urinary ascites

## Abstract

**Background:**

Bladder rupture caused by trauma or pelvic fracture is very common, and can be easily diagnosed. However, Spontaneous rupture of the bladder is rare. Reported by Peters PC. (Peters, Urol Clin N Am 16:279–82, 1989): The incidence of spontaneous bladder rupture is 1: 126000. During childbirth, the occurrence rate of this disease is lower than that of the former. It is very difficult to make an early diagnosis of the spontaneous rupture of the bladder during childbirth, which eventually results in high maternal mortality.

Due to peritoneal reabsorption, the patient may show high levels of serum creatinine and potassium, and this would easily be misdiagnosed as acute renal failure. However, these patients have normal renal function, hence the diagnosis of renal failure is incorrect.

**Case presentation:**

A 23 year-old female patient had her first pregnancy and delivered a full-term healthy baby girl. After delivery, the patient developed fever, oliguria, massive ascites, high serum creatinine and high serum potassium. The patient was initially diagnosed with acute renal failure, however treatment for her condition was ineffective. After further examination, the patient was diagnosed with intraperitoneal bladder rupture. The patient was treated for bladder rupture, made a full recovery and was discharged.

**Conclusions:**

Sudden onset of massive ascites and renal failure due to abnormal serum biochemical characteristics after delivery should be first diagnosed as spontaneous bladder rupture. However, bladder radiography may suggest a false negative result, hence cystoscopy should be performed to confirm the diagnosis. The ratio between ascites creatinine and serum creatinine would be helpful for early diagnosis and to determine the time of rupture. Conservative management or surgical repair should be used to treat bladder rupture.

## Background

Spontaneous bladder rupture is very rarely observed. Reported by Peters PC [1]. The incidence is around 1 in every 126,000 people. There are often underlying causes for spontaneous bladder rupture, of which, tumors, diverticulum and cystitis are the most common. In addition, the uterus of pregnant uterus may increase abdominal pressure to cause spontaneous bladder rupture.

Spontaneous bladder rupture during childbirth has a unique set of clinical symptoms. Patients with severe hematuria could be diagnosed relatively fast, however, those with slight hematuria are often misdiagnosed and have a delayed diagnosis. Fifty-five percent of spontaneous bladder ruptures are intraperitoneal ruptures, and only a few of these would show acute diffusive peritonitis. In these patients, early abdominal pain is often covered by labor pain, and postpartum relaxation of the abdominal wall makes the peritoneal symptoms of bladder rupture not significant, thereby resulting in a delayed diagnosis.

Here, we report a rare case of spontaneous bladder rupture during childbirth.

## Case presentation

The manuscript was approved by the Ethics Committees of Guangdong Women and Children Hosptial and the participant provided written informed consent.

The patient was a 23-year old pregnant woman under healthy condition, and vaginally delivered one healthy girl with term birth as the first pregnancy. Delivery was smooth, first stage of labor was 15 h and second stage was 2 h. The birth weight was 3.6 kg. This newborn was in good health. The slight abdominal bloat was complained after delivery, but without special treatment. The abdominal pain was exacerbated On the fifth day after delivery accompanied with vomiting, shivering, and high fever. The highest body temperature was 39 °C. Oliguria and edema on the bilateral lower extremities were reported. The large amount of ascites was revealed by ultrasound examination; and the venous blood biochemical assays indicated the elevated level of serum creatinine (427 umol/L), urea nitrogen (26 mmol/L) and potassium (6.6 mmol/L). The diagnosis was considered to be postpartum acute renal failure (ARF) and peritonitis The patient was treated with intravenous antibiotics, hemodialysis, peritoneal catheter drainage (2000 mL reddish ascites drained) and indwelling bladder catheter. The abdominal pain was significantly alleviated on the second day and the ascites disappeared with the serum biochemical restored normal. The patient was transferred to our hospital on the sixth day postpartum. Physical examination:Temperature 37.8 °C, Pulse 93 beats per minute, Respiratory rate 30 times per minute, and Blood Pressure 118/78 mmHg. BP118/78 mmHg, Patient was conscious and no abnormality was revealed during heart and lung auscultations. Abdominal distension, mild tenderness and rebound tenderness are positive for the whole abdomen. no percussion pain in the liver and kidney, The bottom of the uterus is at subumbilical 1.5 cm.The intraperitoneal bladder rupture and peri-bladder inflammation were diagnosed and confirmed by MR, CT, cystoradiography and cystoscopy. Exploratory laparotomy was performed to remove the lesion on the bladder wall and repair perforation. The pelvic drainage tube was indwelled. Finally, Patient made a good recovery and was discharged on the 8th day after surgery.

### Intraoperative observation

**Fig. 1:**
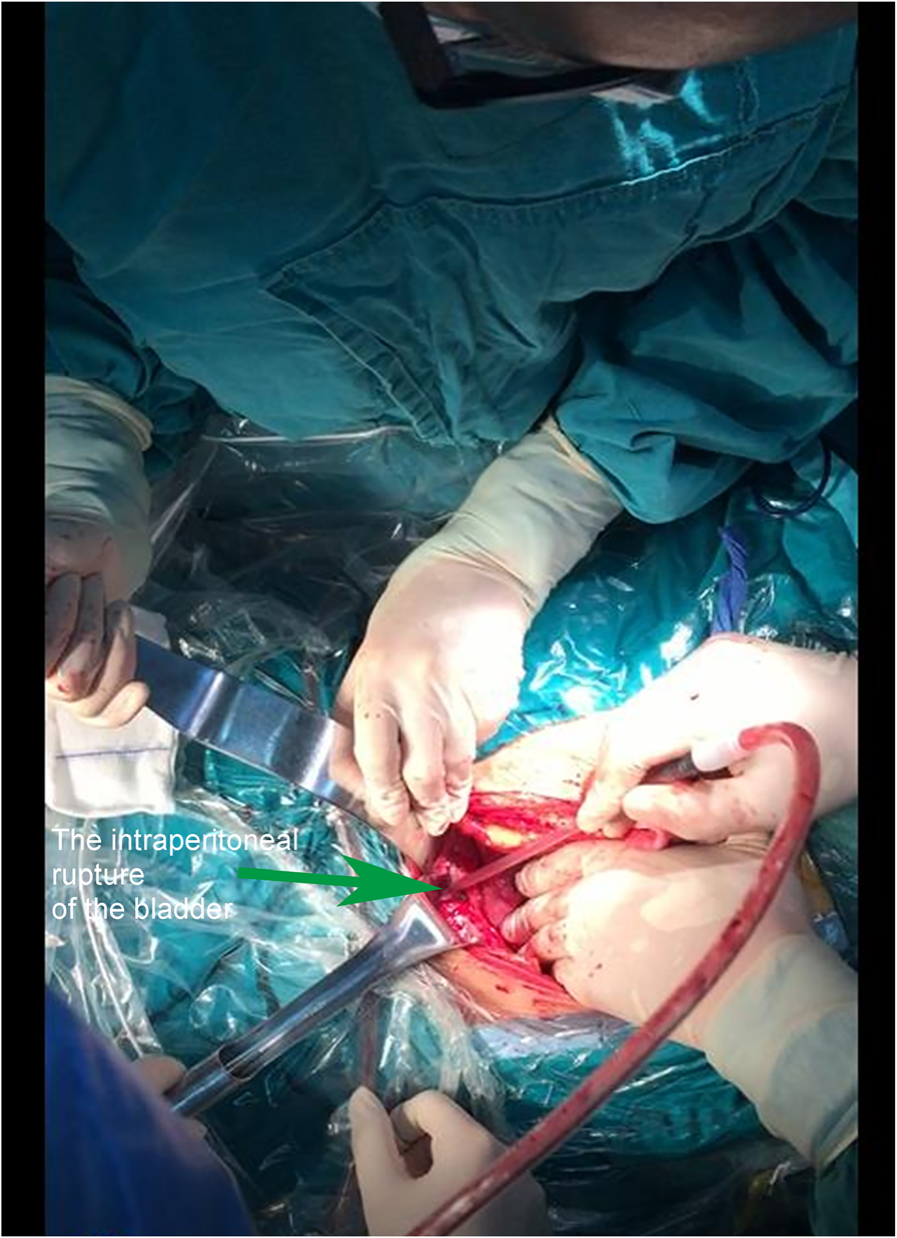
Surgical observation. Intraperitoneal rupture of the bladder was observed, and the breach was irregular

**Fig. 2:**
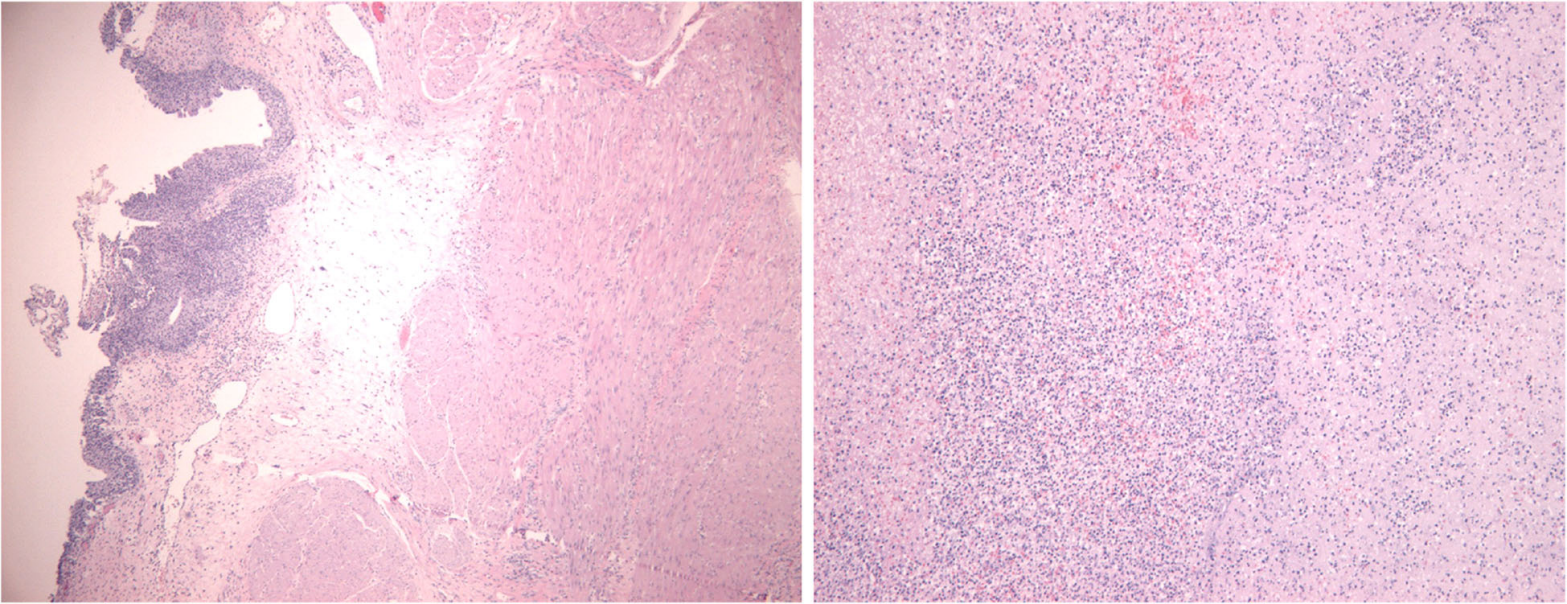
Pathological diagnosis: (bladder). The transitional epithelium under the mucosal lining of bladder tissue. Massive acute and chronic infiltration of inflammatory cells could be seen in parts of the epithelium and mesenchyme. Local hemorrhage and necrosis was also observed. The serosal surface showed infiltration of inflammatory cells and hyperplasia of the granulation tissue. The symptoms are in line with bladder perforation

The bladder was ruptured within abdominal cavity, the rupture site was located on the right top wall of bladder with the size of 10 mm × 10 mm, the edge of rupture was irregular. The local wall of bladder was thickened and formed a mass with size of 60 mm × 30 mm. The mass was located within the serosa membrane and depressed the bladder. The broken site was at the edge of the mass. The edema and adhesion around the bladder was observed (Fig. 1). The mass was proven to be inflammatory granuloma by pathological examination (Fig. 2).

## Discussion

### Diagnostic process

First, the Budd-Chiari syndrome shall be considered for large amount postpartum ascites outburst without obvious induction factor; the therapy is not satisfied with high death rate and poor prognosis. Budd-Chiari syndrome is characterized with venous obstruction on hepatic vein or inferior vena cava at the upstream of hepatic vein, which can be diagnosed by ultrasound examination and angiography of inferior vena cava. The Budd-Chiari syndrome could be excluded for the patient in the current report.

**Fig. 3:**
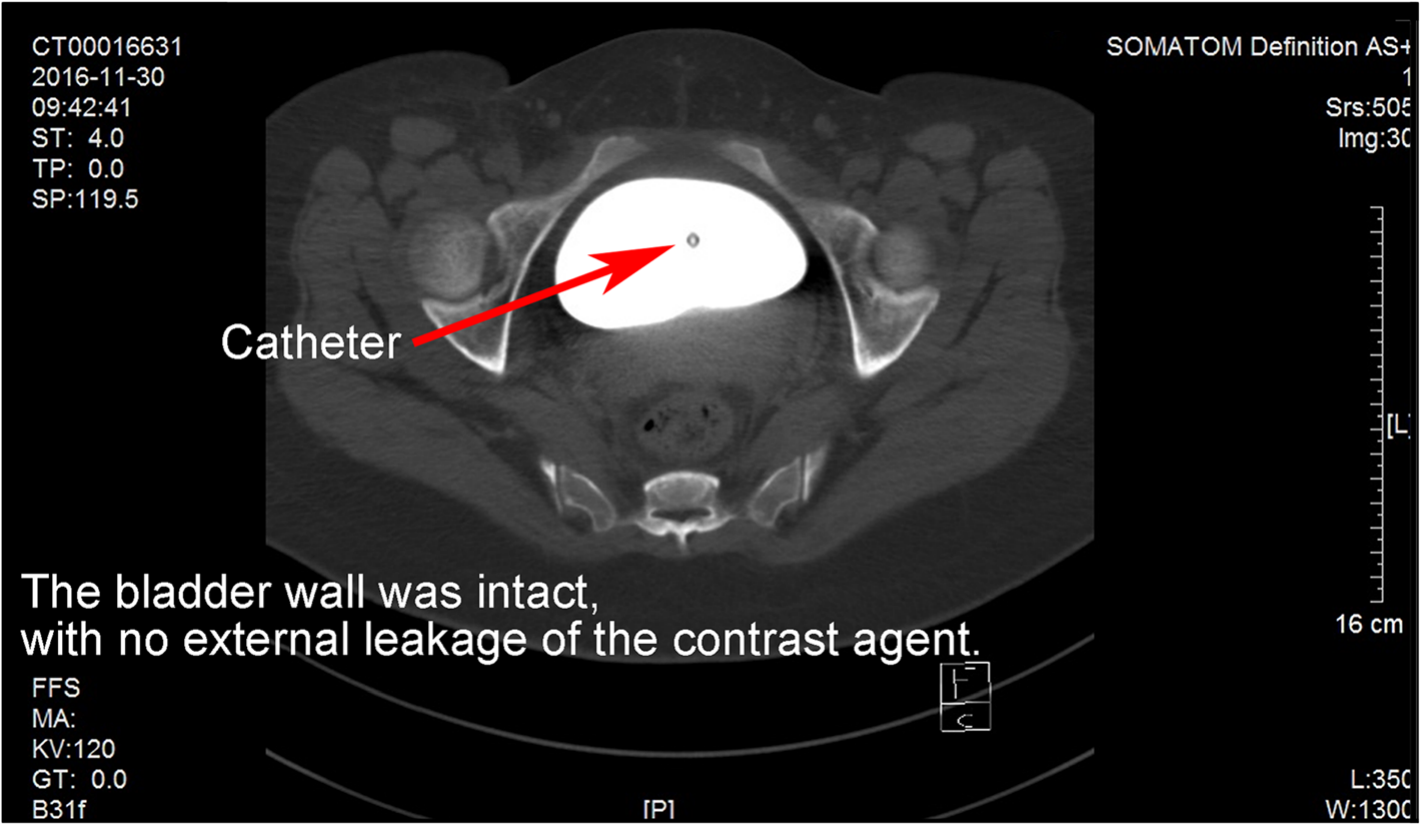
CT bladder radiography. 250 mL of contrast agent was injected via a catheter. The image shows no extravasation of the contrast agent

**Fig. 4:**
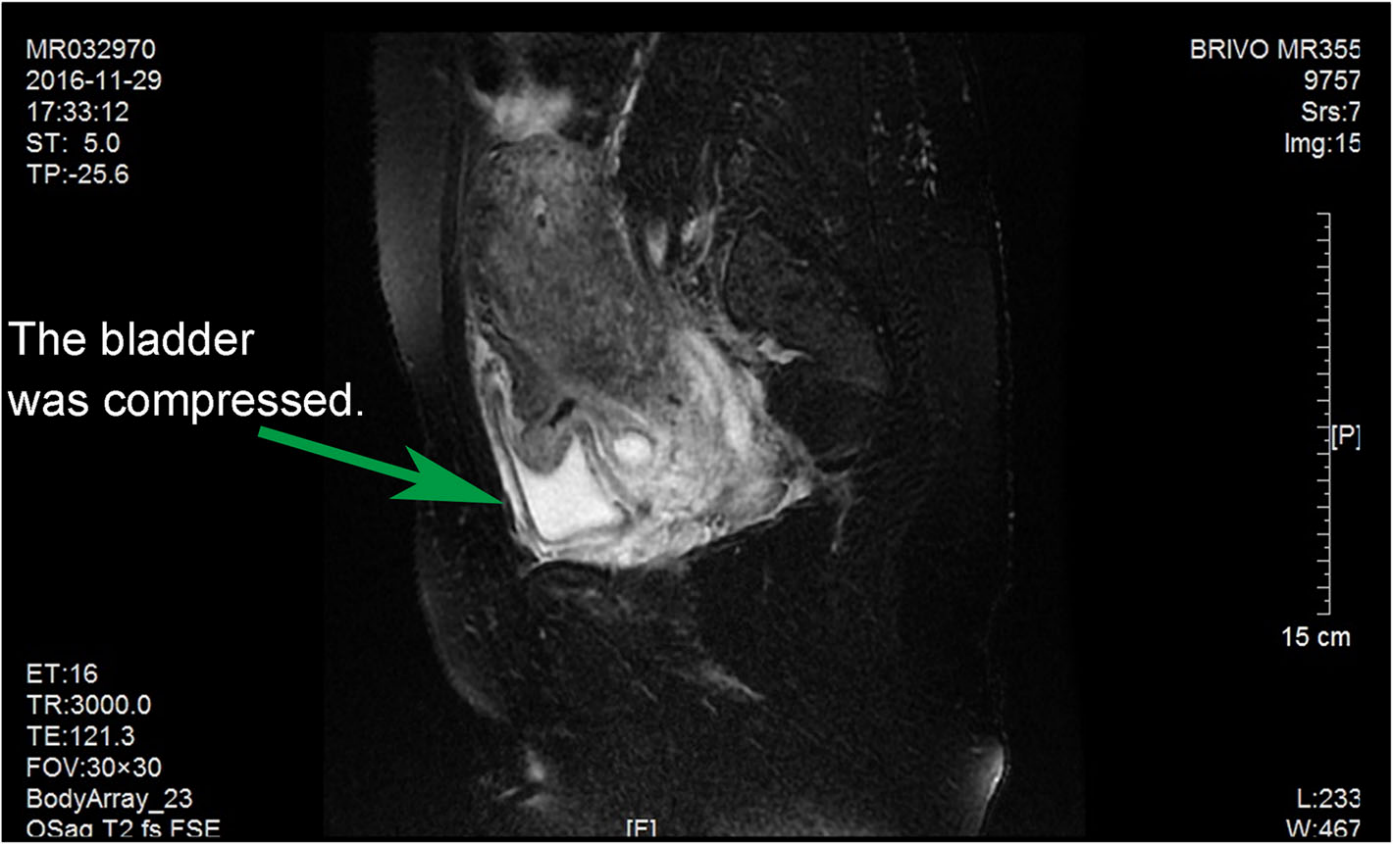
Pelvic MR in the sagittal position. The apex of bladder was observed to be compressed and invaginated

**Fig. 5:**
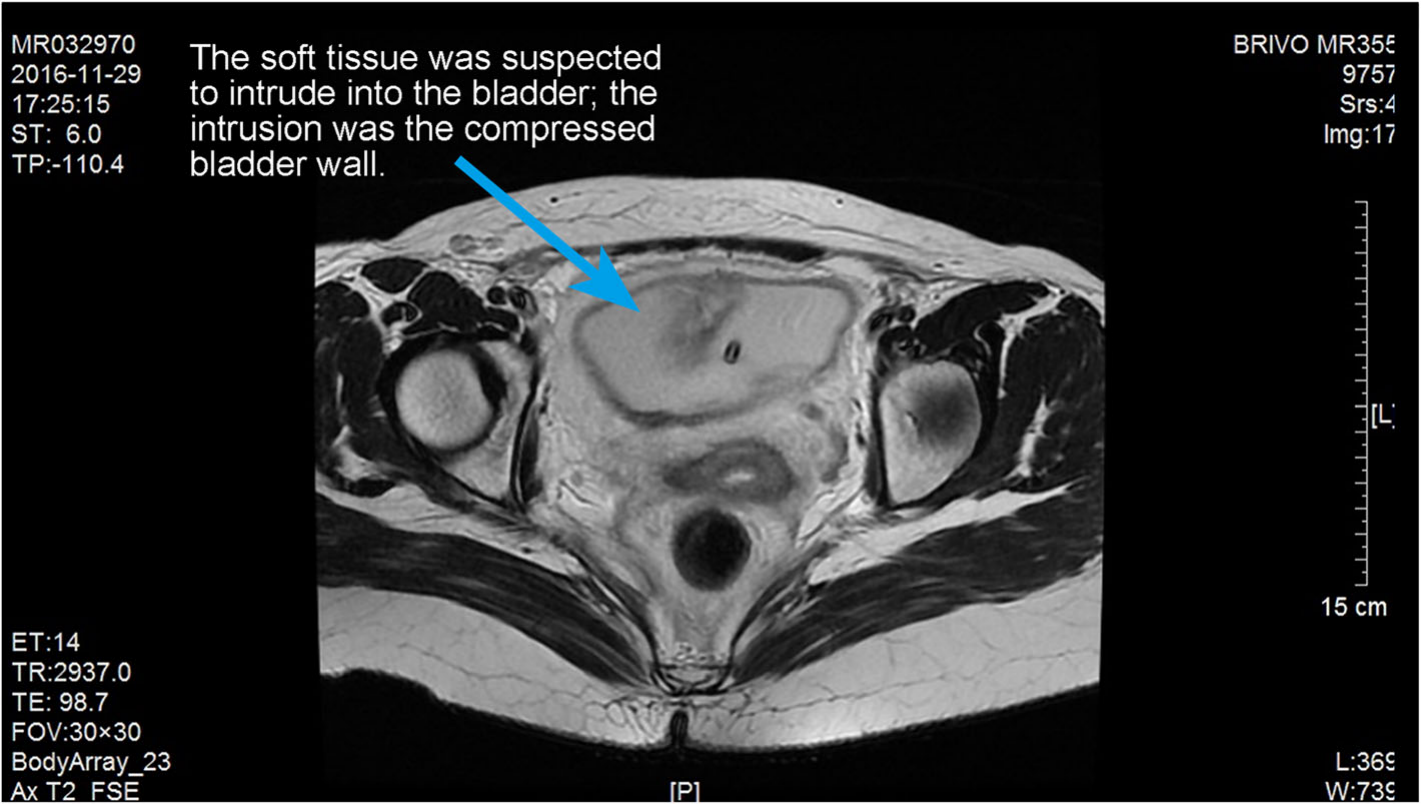
Pelvic MR in the coronal position. The apex of bladder was rough and had wrinkle-like morphology. The shadow observed was suspected to be soft tissue protruding into the bladder cavity (size of 5.8 × 2.9 cm), However, it was later confirmed to be invaginated bladder wall

**Fig. 6:**
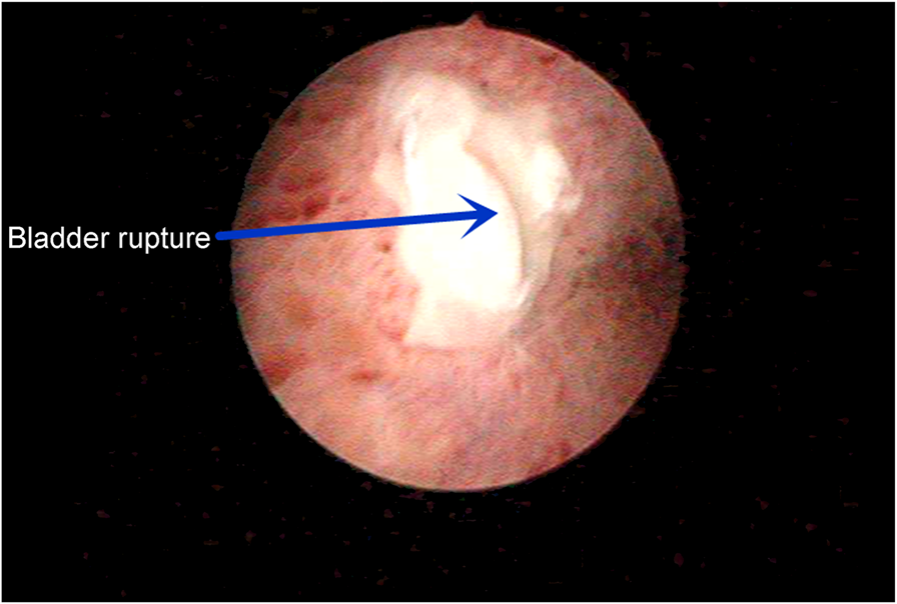
Cystoscopy. The apex of bladder was observed to be ruptured. The rupture was fissure-like, with pale ischemic necrosis in the peripheral tissues

The second, the spontaneous rupture of the bladder was considered. Spontaneous rupture of bladder was very rare and the incidence was reported to be 1:126000 by Peters PC [1]. The potential causes for spontaneous bladder rupture includes bladder neoplasms, bladder diverticulum, and cystitis. In addition, Reported by Heyns CF [2]. The abrupt increase of abdominal pressure and pregnant uterus could induce the bladder rupture as well. Then, the patient underwent CT bladder imaging. We used a catheter to pump the contrast agent 250 ml into the bladder. CT found no extravasation of contrast media (Fig. 3). This result was beyond our expectation. Wirth GJ, reported [3]. The sensitivity and specificity of CT in the diagnosis of bladder rupture were 90 and 100% respectively. Gomez RG, Tonkin JB, Ramchandani P, Arrabal-Polo MA. et al. found that [4–7]. Bladder imaging is the first choice for the diagnosis of bladder injury. The results of CT angiography in this case were very rare. Next, the patient underwent MR examination. MR sagittal film showed the compression of the top wall of the bladder (Fig. 4). The suspected soft tissue projecting into the bladder was found on MR coronal film, and its size was 5.8 ~ 3.9CM (Fig. 5). Based on the MR results, we suspected that the bladder had ruptured and the tissue had blocked the rupture of the bladder, wich resulted in normal CT findings. We decided to perform cystoscopy. Cystoscopy revealed that the top wall of the bladder had been broken. The crevasse was like a crack, and the surrounding tissue was pale and necrotic (Fig. 6). Finally, The patient underwent exploratory laparotomy, The bladder rupture in the abdominal cavity was found. There was a lump on the right side of the top wall of the bladder. The rupture was located at the junction of the bladder mass and the normal tissue (Fig. 1). The pathological diagnosis of the mass is inflammatory granuloma (Fig. 2).

### The etiology

There was a huge inflammatory granulomatous mass in the right wall of the bladder. The wall of the bladder was rigid and the elasticity was poor. When the bladder was filled, the stress of each wall of the bladder was uneven. The sudden increase of abdominal pressure in the delivery period led to the rupture of the bladder.

The rupture is located at the junction of the bladder mass and the normal tissue.

### The cause of delayed diagnosis

**The first**, there was no obvious gross hematuria in the patients. **The second,** there was no diffuse peritonitis at the early stage of bladder rupture. This case developed diffuse peritonitis following postpartum infection on the fifth day of delivery. Reported by Muggia RA, etc [8]. Fifty-five percent of spontaneous bladder rupture was ruptured in the abdominal cavity, and very few patients developed acute diffuse peritonitis. **The third,** perhaps, the pain caused by bladder ruptur was obscured by labor pain, and the bladder pain was ignored. **The fourth,** the patient developed serum biochemical characteristics of renal failure, such as: high levels of serum creatinine and urea nitrogen, hyperkalemia, ascites and so on. Doctors made a diagnosis of the acute renal failure. These factors led to a delay in diagnosis. According to Heyns CF [2]. It is very diffcult to make an early diagnosis of intraperitoneal spontaneous bladder rupture.

The serum and ascites biochemical alterations are helpful for early diagnosis of intraperitoneal spontaneous bladder rupture. Serum levels of creatinine, urea nitrogen and potassium were significantly elevated in the patient, and ascites levels of creatinine as well as urea nitrogen were also increased drastically. It is clinically important to compare the concentration of creatinine in ascites and in serum. Under normal circumstances, ratio of urinary creatinine concentration to serum creatinine concentration is 30: 1~ 100: 1. Since the rupture was located inside abdominal cavity, creatinine in the urinary ascites was reabsorbed by peritoneum The concentration of creatinine in blood was therefore increased.Muggia RA, Rimington PD reported [8]. The ratio of creatinine concentration in ascites to in serums was: 5: 1. In according to Heyns CF, Rimington PD [2]. When diagnosis was delayed more than 24 h, patients will present with above mentioned biochemical characteristics. In this circumstance, intraperitoneal rupture of bladder should be suspected. Although patient’s biochemical characteristics indicated acute renal failure, renal excretory function was normal, therefore the diagnosis of acute renal failure should be carefully avoided.Serum biochemical results at the fifth day after rupture is presented as follow:CR:427 μmol/L,BUN: 26 mmol/L, K:6.6 mmol/L. We are unaware of the clinical significance of ascites creatinine level at the time, the value was not measured.

A conclusive diagnosis could be made cystoscopy or surgical exploration. Radiography cystoscopy was unreliable: false negative finding is likely to be reported due to inadequate filling of cystoradiography, blood clots, obstruction by the surrounding organs, or body position.A false negative image was revealed by CT cystoradiography because of the mass and edema compression around the rupture in this case. A volume of 250 mL of contrast agent was injected into the bladder, but there was no leakage shown (Fig. 3). It was revealed by MR that there was a mass located at the posterior wall of bladder, protruding into the bladder cavity. Pelvic effusion and pericystitis were also found (Figs. 4 and 5). The bladder rupture was confirmed by cystoscopy and the rupture was slit-shaped (Fig. 6). The inflammatory granuloma at the right top of bladder was found by surgical exploration; partial depression on the bladder wall was also revealed with the rupture beside the granuloma, the size was around 10 mm × 10 mm with irregular edge. The peri-bladder inflammatory edema and adhesion were also discovered.

Pathological diagnosis is of great clinical significance. The pathological diagnosis in this case was cystitis granuloma (Fig. 2). Based on pathological diagnosis, we decided the scope of operation: surgical removal of the part of pathological bladder wall and repair of perforation.

Conservative treatment can be used on selected patients with spontaneous bladder rupture. The indication includes that patient is generally well, mild symptom, small perforation, no gross hemorrhage, minimal urinous infiltration, no serious infection, and no intestinal burst into the bladder. If the bladder rupture is caused by any local lesions such as tumor, diverticulum, and inflammatory granuloma, Surgical treatment is suggested. The lesion on the bladder wall should be resected and the rupture repaired with the indwelled pelvic drainage tube. The systematic evaluation and follow up after operation are required. It should be noticed that the bladder wall with residual lesion is the risk of recurrent spontaneous rupture of bladder. Report by Heyns CF, Rimington PD [8]. A case of similar cases was successfully cured by conservative treatment. This patient suffered recurrent spontaneous rupture of bladder, when she got pregnant 2 years later.

Mortality rate of spontaneous rupture of bladder was reported to be 25% by Achraut WH [9]. The patient in this report made a full recovery and was discharged on the 8th day after surgery. There was no abnormality in the follow-up period of 17 months.

## Conclusion

Postnatal sudden onset of massive ascites and abnormal serum biochemical characteristics which resemble renal failure should be firstly considered as spontaneous bladder rupture. Bladder radiography may suggest false negative results, hence cystoscopy should be used to confirm the diagnosis. In addition, biochemical changes to serum and ascites would be helpful for early diagnosis. We recommend surgical exploration for spontaneous bladder rupture on following scenarios: intraperitoneal bladder rupture, acute peritonitis, bladder tumor, etc..
